# BUILDING NETWORKS TO ADDRESS AT-RISK OLDER ADULTS

**DOI:** 10.1093/geroni/igz038.662

**Published:** 2019-11-08

**Authors:** Max Zubatsky, Nina Tumosa

**Affiliations:** 1 Saint Louis University, Saint Louis, United States; 2 Health Resources and Services Administration, Rockville, Maryland, United States

## Abstract

With the rise of older adults and the number of chronic health issues in this population, comes the need for greater collaboration across organizations and health care settings. Age-friendly health systems offer the benefits of providing the best care possible to individuals and families, connect people to specific community resources, and optimize the best access to services and programs. The Gateway Geriatric Workforce Enhancement Program (GWEP) has combined the efforts of Saint Louis University and a rural, critical access hospital to establish a care network across Missouri. Together, this partnership has created a number of services, initiatives, and projects to help older adults maintain independence and offer families ways to take of their loved ones in more effective ways. In this symposium, presenters from Social Work, Marriage and Family Therapy, Geriatric Medicine, Psychology and Nursing disciplines will introduce several areas of this age-friendly network. The four abstracts for this symposium include: 1.) Assessing At-Risk Older Adults through the Rapid Geriatric Assessment, 2.) Cognitive Stimulation Therapy for Individuals with Memory Loss, 3.) Predictors of Falls in Older Adults Across Partner Settings, and 4.) Development of program initiatives such as the Rapid Geriatric Assessment screening, Cognitive Stimulation Therapy, Falls Assessment in Seniors, and Care for Persons with Dementia in their Environments will be covered in detail. At the end of the four presentations, the presenters will highlight the importance of this collaborative network and ways for audience members to consider building an age-friendly network in their community.

